# Quality of diabetes care in patients with schizophrenia: a case-control study in Qatar

**DOI:** 10.1186/s12888-021-03121-5

**Published:** 2021-03-12

**Authors:** Mustafa Abdul Karim, Nadeen Al-Baz, Sami Ouanes, Ali Khalil, Ahmed H. Assar, Abdulkarim Alsiddiqi, Zeinab Dabbous, Mahmoud Zirie, Peter Woodruff, Rayaz A. Malik, Peter M. Haddad

**Affiliations:** 1 Department of Psychiatry, Hamad Medical Corporation, Doha, Qatar; 2 Weill Cornell Medicine-Qatar, Ar-Rayyan, Qatar; 3 Department of Internal Medicine, Hamad Medical Corporation, Doha, Qatar; 4 College of Medicine, Qatar University, Doha, Qatar; 5 Division of Psychology and Mental Health, School of Health Sciences, University of Manchester, Manchester, UK

**Keywords:** Schizophrenia, Diabetes mellitus, Diabetes care

## Abstract

**Background:**

Patients with schizophrenia are at least twice as likely to develop diabetes mellitus compared to the general population. This is of significance in Qatar given the high prevalence of obesity and diabetes. Furthermore, the lifespan of people with schizophrenia is shortened by approximately 15 years, partly due to long-term microvascular and macrovascular complications. High quality diabetes care can significantly reduce morbidity and mortality. We assessed the level of diabetes care delivered to patients in Qatar with schizophrenia and diabetes compared to those with diabetes alone.

**Methods:**

We performed a retrospective chart review of patients with diabetes mellitus with (*n* = 73) and without (*n* = 73) schizophrenia. Demographic information and electronic medical records were reviewed to determine adherence to American Diabetes Association standards of diabetes care in the last 6 and 12 months. Optimal diabetes care was defined as having completed glycated hemoglobin (HbA1c), lipid profile and retinal examination within 12 months.

**Results:**

Optimal diabetes care was significantly lower in patients with schizophrenia and diabetes compared to diabetes alone [26.0% (*n* = 19/73) vs 52.1% (*n* = 38/73), *p* = 0.002]. Patients with diabetes and schizophrenia were also significantly less likely to have had body mass index recorded within 6 months (*p* = 0.008) and HbA1c (*p* = 0.006), lipid profile (*p* = 0.015), estimated glomerular filtration rate (eGFR) (*p* = 0.001) and order for retinal examination (*p* = 0.004) over 12 months. After adjusting for multiple comparisons, only assessment of eGFR (*p* = 0.01) and order for retinal examination (*p* = 0.04) remained significant.

**Conclusion:**

Patients in Qatar with schizophrenia and diabetes, receive sub-optimal diabetes care compared to those with diabetes alone.

## Background

Diabetes Mellitus (DM) has an estimated global prevalence of 8.5% in the adult population [1]. Qatar, Kuwait, and Saudi Arabia in the Middle East and North Africa Region (MENA) are three of the top 10 countries with the highest diabetes prevalence worldwide [2]. In Qatar, 70.1% of Qatari nationals are clinically overweight (Body Mass Index (BMI) ≥25 kg/m^2^), and 41.4% obese (BMI ≥30 kg/m^2^) [3]. The prevalence of diabetes in people with schizophrenia is estimated to be 2 to 3 times higher compared to the general population [4], with over four-fold increased odds of comorbid diabetes in patients with non-affective psychosis and a family history of type II DM [5]. The high prevalence of diabetes in people with schizophrenia reflects multiple factors including a more sedentary lifestyle, higher prevalence of obesity, poorer diet, metabolic side effects of antipsychotic medication and possibly intrinsic factors including genetic predisposition and inflammation [6].

Dysglycemia is also associated with the severity of schizophrenia, particularly negative symptoms and cognitive impairment [7]. A large Danish cohort study investigated diabetes development in antipsychotic-naïve schizophrenia patients [8]. Diabetes was associated with well recognized diabetes risk factors, including higher age, and treatment with a range of antipsychotic drugs. A cross-sectional study in Qatar reported a higher prevalence of metabolic syndrome among psychiatric patients taking antipsychotics versus a control group without psychiatric illness, but the difference did not reach statistical significance (31.9% vs 22.8%, *p* = 0.12) [9]. Most patients in this study were taking second generation antipsychotics and were diagnosed with schizophrenia, schizoaffective disorder or bipolar affective disorder [9]. All-cause mortality for people with schizophrenia is significantly increased, with a recent longitudinal study in the United States showing an all-cause standardized mortality ratio of 3.7 (95% CI, 3.7–3.7) [10]. The lifespan of patients with schizophrenia is shortened by approximately 15 years with the largest single cause of death being cardiovascular disease [11]. Provision of high-quality diabetes care, particularly the management of cardiovascular risk factors, could reduce this elevated mortality and healthcare costs for providers.

Delivering optimal diabetes care in patients with schizophrenia can be challenging as patient, provider, treatment and system-level factors may act as barriers to following evidence-based guidelines [12]. Data on rates of engagement with primary care appointments for patients with schizophrenia are conflicting. A study from Norway found that patients diagnosed with schizophrenia regularly consulted their general practitioner [13] and a cohort study using Danish nationwide registry data showed that people with schizophrenia consulted primary care more frequently compared to age and gender matched controls [14]. However, those with schizophrenia with comorbid medical illnesses had a higher relative risk of not following up with their clinical appointments, 5 years after the index diagnosis [14]. Furthermore, a study from the United States in subjects with diabetes and comorbid psychiatric disorders found that a diagnosis of either bipolar disorder or schizophrenia (versus depression) was significantly associated with decreased appointment attendance [15]. Although, after multivariate analysis and adjustment, only bipolar disorder remained associated with reduced clinic attendance [15]. 

Several studies have reported that patients with DM and serious mental illness (SMI) are less likely to receive optimal diabetes care [12, 15–17]. In Canada, Kurdyak et al. (2017) showed that schizophrenia was associated with a reduced likelihood of optimal diabetes care (HbA1c, lipid profile and retinal examination) over the preceding 2 years [adjusted odds ratio (OR) (95% CI): 0.64 (0.61–0.67)] and an increased likelihood of diabetes-related emergency department (ED) visits (adjusted OR (95% CI): 1.34 (1.28–1.41)) and hospitalization (adjusted OR (95% CI): 1.36 (1.28–1.43)) [12]. These results are consistent with an earlier study showing an increased rate of diabetes-related emergency department visits in people with schizophrenia (Hazard Ratio = 1.74, 95% CI (1.42–2.12)) [18]. Indeed, the non-treatment rates for diabetes in the Clinical Antipsychotic Trials of Intervention Effectiveness (CATIE) study in patients with schizophrenia was 45.3% [19]. However, a study by Whyte et al in 2007 found that the quality of diabetes care in the United Kingdom was comparable between patients with schizophrenia or bipolar disorder with comorbid diabetes and those with diabetes alone [20]. This contrasting result may reflect the fact that Whyte et al. examined the quality of diabetes care in the first year following the introduction of a new government contract for primary care doctors which offered financial incentives if specific targets were met. These included 17 quality indicators for diabetes care. Indeed, engagement with mental health services and adherence to antipsychotic treatment is associated with a significant reduction in diabetes related hospitalization [21]. An association has also been found between more frequent outpatient psychiatry (OR = 1.28, 95% CI = 1.20–1.37) and primary care (OR = 2.10, 95% CI = 1.85–2.39) visits and optimal diabetes testing [22].

The quality of diabetes care received by people with schizophrenia in Qatar has not been investigated previously. This is of relevance given the high prevalence of obesity and diabetes in Qatar. Our aim was to determine the quality of diabetes care in patients with schizophrenia and diabetes compared to patients with diabetes alone.

## Methods

### Setting

Qatar is situated on the west coast of the Persian Gulf. Nearly 90% of the country’s population of 2.9 million people are expatriate workers. Most of the population live in Doha, the capital. The median age is 32 years. Hamad Medical Corporation (HMC) and the Primary Health Care Corporation (PHCC) are the main providers of free or subsidized governmental healthcare services in The State of Qatar. HMC provides comprehensive secondary and tertiary level care that includes mental health outpatient clinics and diabetes clinics as well as inpatient care. The PHCC provides primary care services via modern primary care health centers. Electronic Medical Records (EMRs) include a list of all encounters within HMC and PHCC for each patient. Our population of interest included all patients being followed up with mental health outpatient services and/or the diabetes clinic at HMC.

### Period of study

Data were extracted retrospectively from EMRs between January and December of 2018.

### Eligible population and sample

We identified two cohorts of patients on the CERNER Electronic Medical Records (EMRs) system in outpatient clinics in Hamad Medical Corporation (HMC), Doha, Qatar: those with diabetes and schizophrenia (study group) and those with diabetes and no serious psychiatric illness (control group). Cohorts were limited to subjects 18 years of age and older. Both genders and all nationalities were included. Diagnoses of both diabetes and schizophrenia were based on documentation on EMRs. Standards of diabetes care were retrospectively recorded for the year 2018, and patients were only included if they were diagnosed with both DM and schizophrenia prior to 2018. Patients were assigned subject numbers and selected using a random number generator.

### Variables included

Based on recommendations by the American Diabetes Association (ADA) [23], we used the completion of the following as indices of optimal diabetes care: HbA1c, BMI and blood pressure over 6 months (July through December 2018) and HbA1c, BMI, blood pressure, lipid profile, retinal examination, foot examination, serum creatinine and eGFR over 12 months (January through December of 2018). In addition to the ADA key measures of optimal diabetes care, we added referrals to the dietician and diabetes educators as two additional indices since these are recommended by the local guidelines and are key to the implementation of changes to the management of patients with diabetes. We also recorded the total number of medical and psychiatric admissions over 12 months.

To define optimal diabetes care as a binary variable, we chose the completion of HbA1c, lipid profile, and retinal examination within 12 months. This definition was used in a previous study by Kurdyak et al [12].

### Data sources

Patient demographics, encounters with different facilities and specialties, in addition to investigations, diagnostic and therapeutic procedures are all currently available on EMRs. We extracted sociodemographic and clinical data from EMRs and recoded the data in a computerized data base for analysis.

### Sample size calculation

A previous study showed that the rate of HbA1c testing in patients with schizophrenia and DM was significantly lower compared to patients with diabetes alone (48% vs 71%, *p* = .0001) [24]. Using the same proportions, with an alpha = 0.05, power = 0.9, and sample size distribution ratio of 1:1, the calculated sample size in each group was 73, with a total sample size of 146 [25].

### Statistical analysis

Statistical analysis was conducted using the Statistical Package for the Social Sciences (IBM-SPSS, version 23.0, IBM Corp, Armonk, NY, USA). For categorical variables, we calculated the frequency. For continuous variables, we calculated the mean and standard deviation (SD). The differences between the study and control groups were analyzed using Pearson’s chi-squared test for categorical variables (and in case of non-validity, the Fishers’ exact test), and t-test for continuous variables. For multiple comparisons, *p* values were adjusted using the Holm Bonferroni method.

### Ethical issues

The study was approved by the Medical Research Center (MRC) and Institutional Review Board (IRB). The IRB confirmed that patient consent was not required as the data was obtained from existing medical records. The study complied with all HMC research governance protocols.

## Results

### Sociodemographic and clinical characteristics of the groups

The study assessed participants with schizophrenia and diabetes (*n* = 73) versus those with diabetes only (*n* = 73). Participants with diabetes and schizophrenia were significantly younger compared to the control group (45.1 ± 13.0 vs 57.9 ± 11.1; *p* < 0.001). There was no significant difference between the two groups in terms of gender, nationality, BMI, HbA1c and the number of admissions (medical and psychiatric combined) (Table 1).

**Table 1:** Sociodemographic and clinical characteristics of patients with diabetes vs patients with diabetes and schizophrenia

	Diabetes (***n*** = 73)	Diabetes + Schizophrenia (***n*** = 73)	***p***
**N**	73	73	
Age, years	57.9 ± 11.1	45.1 ± 13.0	0.001
Gender, % male	57.5	58.9	0.500
Nationality			
Qatari, %	46.6	60.3	0.063
Non-Qatari Arab, %	24.7	19.2	
South Asian, %	23.3	9.6	
Other, %	5.4	10.9	
Last BMI, Kg/m^2^	32.6 ± 6.1	32.1 ± 7.5	0.690
Last HbA1c, %	8.1 ± 1.8	7.4 ± 2.3	0.055
Total number of admissions over the last year (medical and psychiatric combined) for each group	11	16	0.286

### Standards of optimal diabetes care

Five standards were significantly less likely to be met in patients with diabetes and schizophrenia compared to diabetes alone: BMI recorded within 6 months (*p* = 0.008) and HbA1c (*p* = 0.006), lipid profile (*p* = 0.015), eGFR (*p* = 0.001) and order for retinal examination over 12 months (*p* = 0.004). However, after adjusting for multiple comparisons, only assessment of eGFR (*p* = 0.01) and order for retinal examination (*p* = 0.04) within the last 12 months remained significant (Table 2). Using the three-item binary definition of optimal diabetes care (HbA1c, lipid profile and retinal examination over 12 months), patients with diabetes and schizophrenia were less likely to receive optimal diabetes care compared to those with diabetes alone [26.0% (*n* = 19/73) vs 52.1% (*n* = 38/73), *p* = 0.002].

**Table 2:** Standards of optimal diabetes care and monitoring in patients with diabetes vs patients with diabetes and schizophrenia

	Diabetes (***n*** = 73)	Diabetes + Schizophrenia (***n*** = 73)	***p***	Adjusted ***p***
BMI recorded in the last six months, %	72.6	52.1	0.008	0.064
BP recorded in the last six months, %	95.9	94.5	0.500	1.000
HbA1c obtained in the last six months, %	72.6	63.0	0.144	0.567
HbA1c obtained in the last 12 months, %	84.9	65.8	0.006	0.054
Lipid panel obtained in the last 12 months, %	84.9	68.5	0.015	0.105
eGFR obtained in the last 12 months, %	89.0	67.1	0.001	0.011
Retinal examination ordered in the last 12 months, %	57.5	34.2	0.004	0.040
Foot examination ordered in the last 12 months, %	17.8	17.8	0.585	1.000
Referred to diabetes educator in the last 12 months, %	17.8	9.6	0.114	0.576
Referred to dietitian in the last 12 months, %	30.1	17.8	0.060	0.360

*BMI* Body Mass Index, *BP* Blood pressure, *eGFR* estimated glomerular filtration rate; *p* values were adjusted using the Holm Bonferroni method

Data expressed as Mean ± Standard Deviation; *BMI* Body Mass Index

## Discussion

After controlling for multiple testing, we found that patients with diabetes and schizophrenia were significantly less likely to meet 2 ADA standards of diabetic care compared to patients with diabetes alone. These were assessment of eGFR and order for retinal screening examination within the last 12 months. Using the three-item definition of optimal diabetes care (HbA1c, lipid profile and retinal examination over 12 months), patients with diabetes and schizophrenia were half as likely to receive optimal diabetes care compared to those with diabetes alone. A Canadian study using the same definition over 2 years reported very similar findings [12]. Our findings are consistent with a series of previous studies wherein patients with schizophrenia are less likely to receive high quality diabetes care [15–17].

Suboptimal diabetes care in our study group may be attributed to a lack of integrated physical and mental health services and difficulty sharing information and expertise amongst health care specialties [26]. Stigma and lack of training for non-psychiatric physicians on the physical and mental health care needs for people with SMI might further hinder optimal health care delivery. Magliano el al (2017) found that general practitioners (GPs) perceived patients with schizophrenia as dangerous, less likely to recover, and in need of long-term pharmacotherapy [27]. Fleury et al. (2012) found that GPs in Quebec regarded themselves as competent managing common but not serious mental health problems. Over half of participating GPs reported no contact with mental health resources, and most regarded the mental health system to be of poor quality, particularly in terms of accessibility and availability of services [28]. Symptoms of schizophrenia, including psychotic symptoms, negative symptoms, cognitive deficits and impaired insight have been implicated in poor adherence to medication [29] and clinic appointments. Schizophrenia is also frequently comorbid with depression and substance misuse [30], which can further hinder engagement with diabetes care. Socioeconomic status is unlikely to affect the quality of care in Qatar, as health care services are mostly free of charge for citizens and provided with minimal charge for residents of the country.

In our sample, patients with diabetes alone were significantly older compared to those with both diabetes and schizophrenia, which could reflect the earlier onset of diabetes due to lifestyle and medications used for schizophrenia [8]. However, the level of obesity (BMI) and glycemic control (HbA1c) was comparable between the two groups. A recent study in Qatar also found no significant difference in central obesity or the prevalence of metabolic syndrome between patients receiving antipsychotics and controls [9]. The prevalence of obesity, metabolic syndrome, and glucose abnormalities was high in both groups. This possibly indicates either a type II error or the fact that the high prevalence of obesity and metabolic syndrome among the general Qatari population might have masked any further effect of antipsychotics or schizophrenia on these outcomes.

We propose several strategies to reduce the inequalities reported. Educational awareness programs for health professionals are needed to address mental health stigma and negative stereotypes. Further work is required to increase awareness amongst HCPs regarding the increased morbidity and mortality in patients with schizophrenia. Communication between different services providing health care for patients with schizophrenia is essential to ensuring access to health care services and optimizing management of medical comorbidities. Education to prevent or limit obesity and diabetes needs to be promoted among patients with severe mental illness to help decrease the prevalence of diabetes and ensure that those diagnosed with diabetes receive high quality care. These recommendations are in line with the recently published blueprint for protecting physical health in people with mental illness [31].

### Strengths and limitations

This population-based study included randomly assigned patients from both genders and all nationalities followed in outpatient clinics. Data to identify and control for the duration of diabetes and schizophrenia in our cohorts was limited as the EMRs system was started in 2015. Additionally, important demographic information such as smoking, marital and socioeconomic status were missing or inconsistently documented. Nevertheless, lab results, orders and documentation pertaining to diabetes care were easily extractible, and the criteria for optimal diabetes care were clearly defined. As this is a case-control study, the incidence and relative risk of suboptimal diabetes care in patients with schizophrenia could not be calculated.

## Conclusion

This study found that patients with schizophrenia and diabetes in Qatar are less likely to receive optimal diabetes care compared to patients with diabetes alone, particularly in relation to the assessment of microvascular complications with a decreased frequency of eGFR assessment and request for retinal examination. These data demand further larger population cohort studies to assess adherence with treatment and the burden of diabetes-related medical comorbidities and complications in patients with schizophrenia.

## Data Availability

The data that support the findings of this study are available from the corresponding author upon reasonable request and pending additional ethical approval.

## Abbreviations

ADA: American Diabetes Association
BMI: Body-Mass Index
CATIE: Clinical Antipsychotic Trials of Intervention Effectiveness
CVD: Cardiovascular Disease
CI: Confidence Interval
DM: Diabetes Mellitus
ED: Emergency Department
eGFR: Estimated Glomerular Filtration Rate
EMRs: Electronic Medical Records
GPs: General Practitioners
HbA1c: Glycosylated Hemoglobin
HCPs: Health Care Professionals
HMC: Hamad Medical Corporation
IRB: Institutional Review Board
MENA: Middle East and North Africa
OR: Odds Ratio
SMI: Serious Mental Illness
